# Supplementary material for: Resolving the full spectrum of human genome variation using Linked-Reads
Source: Genome Res. 2019 Apr;29(4):635–45. doi: 10.1101/gr.234443.118 (PMC6442396; doi:10.1101/gr.234443.118)
Supplement: Supplemental Material [file supp_gr.234443.118_Supplemental_longranger-2.2.zip › longranger-2.2.tar.bz2!/lr2.2-public/lib/stan/cmdstan/stan/doc/api/html/multivariate_2continuous_8hpp_source.html]

Stan: src/stan/prob/distributions/multivariate/continuous.hpp Source File


|  |  |
| --- | --- |
|  | Stan  2.5.0  probability, sampling & optimization |


- Main Page
- Related Pages
- Namespaces
- Classes
- Files

- File List
- File Members

All Classes Namespaces Files Functions Variables Typedefs Enumerations Enumerator Friends Macros Pages

- src
- stan
- prob
- distributions
- multivariate

continuous.hpp

Go to the documentation of this file.

1 #ifndef STAN\_\_PROB\_\_DISTRIBUTIONS\_\_MULTIVARIATE\_\_CONTINUOUS\_HPP

2 #define STAN\_\_PROB\_\_DISTRIBUTIONS\_\_MULTIVARIATE\_\_CONTINUOUS\_HPP

3

4 #include <stan/prob/distributions/multivariate/continuous/gaussian\_dlm\_obs.hpp>

5 #include <stan/prob/distributions/multivariate/continuous/matrix\_normal.hpp>

6 #include <stan/prob/distributions/multivariate/continuous/multi\_normal.hpp>

7 #include <stan/prob/distributions/multivariate/continuous/multi\_normal\_cholesky.hpp>

8 #include <stan/prob/distributions/multivariate/continuous/multi\_normal\_prec.hpp>

9 #include <stan/prob/distributions/multivariate/continuous/multi\_gp.hpp>

10 #include <stan/prob/distributions/multivariate/continuous/multi\_gp\_cholesky.hpp>

11 #include <stan/prob/distributions/multivariate/continuous/multi\_student\_t.hpp>

12 #include <stan/prob/distributions/multivariate/continuous/wishart.hpp>

13 #include <stan/prob/distributions/multivariate/continuous/inv\_wishart.hpp>

14 #include <stan/prob/distributions/multivariate/continuous/dirichlet.hpp>

15 #include <stan/prob/distributions/multivariate/continuous/lkj\_corr.hpp>

16 #include <stan/prob/distributions/multivariate/continuous/lkj\_cov.hpp>

17

18 #endif

multi\_gp\_cholesky.hpp

multi\_normal\_prec.hpp

lkj\_corr.hpp

multi\_normal\_cholesky.hpp

gaussian\_dlm\_obs.hpp

wishart.hpp

matrix\_normal.hpp

dirichlet.hpp

lkj\_cov.hpp

multi\_gp.hpp

multi\_normal.hpp

multi\_student\_t.hpp

inv\_wishart.hpp


---

[ Stan Home Page ]

*© 2011–2014,
Stan Development Team.*
